# Impact of malaria related messages on insecticide-treated net (ITN) use for malaria prevention in Ghana

**DOI:** 10.1186/1475-2875-13-123

**Published:** 2014-03-28

**Authors:** Ebenezer S Owusu Adjah, Andrie G Panayiotou

**Affiliations:** 1 Cyprus International Institute for Environmental and Public Health in Association with the Harvard School of Public Health, Cyprus University of Technology, Limassol, Cyprus

**Keywords:** ITN, Insecticide treated nets, Ghana, Bed net, Media messages, Household

## Abstract

**Background:**

Media messages have been used in Ghana to promote insecticide-treated net (ITN)/bed net usage in an effort to impact on malaria prevention. The aim of this study was to assess the effect of such malaria-related messages delivered through electronic/print media and by volunteers/health workers on the use of ITNs by children living in a household.

**Methods:**

Data was collected from September to November of 2008 using a structured, interviewer-administered questionnaire by the Ghana Statistical Service as part of a national demographic and health survey (DHS). Secondary data analysis was performed on the collected data using multivariate logistic regression for both individual messages and a composite (any of) message variable.

**Results:**

From the 11,788 households surveyed, 45% had at least one net. Households with male heads were more likely to have a child sleeping under a bed net the previous night (p = 0.0001). Individual Messages delivered by a health worker or a dedicated radio programme, had the highest effect for one or more children sleeping under a net the night before (OR _adjusted_ = 1.65; 95% CI = 1.44 to 1.88 and OR _adjusted_ = 1.26; 95% CI =1.12 to 1.42 respectively) while hearing any of the eight messages (composite score) resulted in the highest odds for one or more children (OR _adjusted_ = 3.06; 95% CI = 2.27 to 4.12) sleeping under a bed net.

**Conclusion:**

Efforts to relate ITN messages to the public are very useful in increasing use of bed nets and having multiple ways of reaching the public increases their effect, with the biggest effect seen when health workers and volunteers were used to deliver malaria-related messages to the public.

## Background

As a malaria endemic country, Ghana records about 3.2 million malaria cases annually with about 38,000 of these cases leading to death [1], thus making the need for better prevention and control of malaria cases imperative. Malaria prevention and control is best practiced as an integrated approach involving first-line drugs, case management, indoor residual spraying (IRS) and the use of insecticide-treated nets (ITN). The use of ITN and indoor residual spraying is recommended for all persons at risk [1] and the impact of ITNs on malaria control has been sufficiently documented with reports that population coverage of ITN of over 70%, reduces clinical malaria and all-cause mortality in children by 15% to 30% in Nigeria [2]. Additional studies have shown an estimated 33% reduction in mortality among children with the use of treated nets in Kenya [3] and a 17% reduction in childhood mortality due to ITN use in Ghana [4].

Various communication strategies have been implemented so far in order to promote the prevention and control of malaria at both the community [5] and the individual level [6]. Ghana officially adopted the use of treated nets as a vector control programme in 2004 [7] and with the help of The Global Fund to Fight AIDS, TB, and Malaria, The US President’s Malaria Initiative (PMI) and the World Bank, about 2.4 million procured ITNs were distributed to Ghanaian households in 2007, which led to about 30% of households in Ghana owning a net [1]. Despite the fact that this number is much lower than the number of total households in Ghana, education programmes by the Ministry of Health and NGO’s aimed at promoting the use of ITNs have complemented the free distribution of nets. However, given that the number of outpatient malaria cases reported in 2007 and 2008 (i.e. after the distribution of malaria nets) has not been reduced accordingly [1], it is unclear whether individuals within households that own an ITN actually use it at night and whether malaria related education programmes are actually effective.

Thus this study aimed to assess the effect of malaria related messages delivered through the electronic/print media and by volunteers/health care workers on the use of ITNs by children, using data from the 2008 demographic and health survey (DHS) in Ghana. Specifically, the effect of TV, radio, newspaper, poster, health worker and community volunteer related malaria prevention messages on the use of ITN by children living in a household was assessed.

## Methods

### Data source

Data from the household questionnaire of the 2008 Ghana Demographic and Health Survey (DHS) was used in this study. The DHS survey was originally designed to allow reliable estimation of key demographic and health indicators, such as fertility, contraceptive prevalence, nutritional status, infant and child mortality and anaemia prevalence. Funded by the United States Agency for International Development (USAID), it was conducted by the Ghana Statistical Service in collaboration with MACRO International [8]. This study was a nationally representative survey that sampled about 12,000 households using a weighted approach. Specifically, ten administrative regions were subdivided into districts and later into localities. Localities were then subdivided into enumeration areas, two or three of which became the sampling unit from which the households were randomly selected [8]. In total, 11,788 households had complete information and contributed towards the household level estimates. According to the DHS, a household is: “a person or group of persons that usually lives and eats together” [9]. Therefore, a household includes all people who live together, whether they are related or not and the survey respondent (irrespective of whether he/she is the head of household) was the one who answered household questions regarding exposure to the messages as well as the number of children (under five years) who slept under the net the previous night. Access to demographic and health survey data is managed and provided by MEASURE DHS following an online registration [10].

From the data available, the main outcome: “number of children (under 5 years) who had slept under a net the previous night”, obtained by an interviewer-based questionnaire given to the representative of the household was extracted. Bed nets were defined as all long-lasting insecticide-treated and other insecticide-treated nets (ITN). Therefore, all insecticide- treated nets, whether long-lasting insecticide-treated nets or re-treated nets are referred to here as net or bed net. With regards to malaria related media messages, the survey had obtained information on TV, radio, newspaper, poster, health worker and community volunteer delivered messages by asking the household representative the question: “in the past 12 months, have you seen or heard any messages telling you families should sleep under ITN, especially pregnant women and children on: (i) radio, (ii) TV, (iii) newspaper, (iv) poster, (v) leaflet or brochure, (vi) by health worker, (vii) community volunteer, and (viii) “Hehaho”. “Hehaho” stands for “Healthier Happier Home” and it is a radio serial drama show, designed to educate the public on childhood diseases and family health issues at home [11].

Furthermore, information on the sex of the household head, type of place of residence (rural or urban), whether the household had any type of net (yes or no), number of nets in the household (none, 1–2 nets, 3 or more nets), household size (calculated from the number of individual members per household and coded as 0, 1 and 2 for 1–4, 5–9 and 10+ members respectively) and the household’s wealth index, constructed by using information on household ownership of consumer items, ranging from a television to a bicycle or car, as well as dwelling characteristics, such as source of drinking water, sanitation facilities, and type of flooring material as described in Ghana Demographic and Health Survey, 2008 [8].

### Statistical methods

DHS uses a multistage cluster sampling approach with households nested within communities [8,12], so the SURVEY procedures in SAS which incorporates sampling weights to account for sampling design was used for the analysis. Outcomes were coded as 0/1 for households that had answered “no child/1 or more children” to the question of “number of children who slept under a net the previous night”. Malaria-related messages (i.e. exposures) were also binary (no/yes). In addition, a composite message variable was created based on whether a household had heard any of the eight types of messages (no/yes).

Associations between messages in the past 12 months and having a child sleep under a net were investigated by computing age and sex of the household head-adjusted (crude) and fully-adjusted (adjusted for age of head of household, sex of head of household, type of place of residence, household size and wealth index) odds ratios using logistic regression. These confounders were tested first for association with both the exposures and the outcome using chi-square for categorical variables and student’s *t*-test for continuous variables. Where necessary a chi-square test for trend was performed for the categorical variables with more than 2 levels (e.g. wealth index). Preliminary model diagnostics revealed the absence of multi-colinearity of the predictor variables (source of malaria related messages) measured. In addition to adjusting for confounders, cross exposure was also accounted for by including all sources of messages in the model building process. This way, the effect estimates obtained would be a true reflection of exposure to a particular message and not a combination of a number of messages. The number of nets owned by each household was not adjusted for in the analysis as it is in the causal pathway (prerequisite) between hearing a relevant message and using a bed net (effect). Effect modification by sex of household head and type of place of residence was also tested by adding the interaction terms into the models.

Data were analysed using SAS version 9.2 (SAS Institute). All tests were two tailed and a p < 0.05 was considered statistically significant. Missing data was less than 0.3% for all variables.

## Results

### Baseline characteristics of households surveyed

Of the 11, 778 households surveyed, 5,175 (47.8%) were in urban areas and 6,603 (52.2%) in rural areas. These households were made up of 44,556 individual members (~8.6 members/household). It is encouraging that 45% of households had at least one net and 40% had 1–2 nets. Men were household heads in 66.3% of households (Table 1). Rural households were also more likely to have any child sleep under a net compared to urban households (67.2% vs. 32.8%, p = 0.0001) (see Additional file 1). Of all the households surveyed, survey respondents in 9, 428 (81.4%) households reported having heard a malaria message on the radio making this category of messages the most far-reaching. The lowest medium through which households had heard a malaria message in the past twelve months was leaflets, with only 1, 310 (12.0%) households reporting having read a malaria message from leaflets (see Additional file 1).

**Table 1:** Socio-demographic characteristics of the participating households (n = 11 778)

**Survey characteristic**	
	**Mean ± SE**
**Age of household head**	44.39 ± 0.15
	**N (%)**
**Household size**	
1–4 members	7479 (65.9)
5–9 members	3906 (31.5)
10+ members	393 (2.6)
*p-value	< 0.0001
**Type of place of residence**	
Rural area	6603 (52.2)
Urban area	5175 (47.8)
*p-value	< 0.0001
**Sex of household head**	
Male	8043 (66.3)
Female	3735 (33.7)
*p-value	< 0.0001
**Household wealth index**	
Poorest	2490 (15.4)
Poorer	2291 (19.1)
Middle	2354 (21.6)
Richer	2402 (22.5)
Richest	2241 (21.4)
*p-value	< 0.0001
**Household has at least one mosquito net**	
Yes	5652 (45.4)
No	6125 (54.6)
*p-value	< 0.0001
**Number of nets in household**	
none	6126 (54.6)
1–2 nets	4795 (39.8)
3 or more nets	857 (5.7)
*p-value	< 0.0001

*p-value for the differences between distribution of demographic subgroups using chi-square test for proportions.

The potential confounders tested (age and sex of household head, type of place of residence, wealth and household size) were strongly associated with all media messages and the outcome under study (see Additional file 1) and were, therefore, adjusted for in the analysis.

### Association between media messages and children sleeping under a net

Table 2 shows the associations between malaria messages and children sleeping under a net the previous night. After adjusting for age and sex of head of household, household size, type of place of residence and wealth index, households that had heard malaria messages on the radio, from community volunteer, health worker and the “Hehaho” programme were associated with higher likelihood of net use by children under five (Table 2). Having heard a malaria message from a health worker or the dedicated radio programme “Hehaho”, was associated with significantly increased odds of one or more children sleeping under a bed net (OR _adjusted_ = 1.65; 95% CI = 1.44 to 1.88 and OR _adjusted_ = 1.26; 95% CI =1.12 to 1.42 respectively) (Table 2). Notably, having heard any of the eight messages (composite score) resulted in the highest odds for one or more children sleeping under a bed net (OR _adjusted_ = 3.06; 95% CI =2.27 to 4.12). Restricting analysis to only households with children under five years of age did not significantly change the effect estimates (see Additional file 2).

**Table 2:** Associations between malaria related messages and number of children sleeping under a net the previous night

**Source of malaria message**	**Number of children who slept under a net (≥ 1 child vs. No child**)					
	Crude OR*	**95% CI**	**p-value**	**Adjusted OR**^**†**^	**95% CI**	**p-value**
**Any (composite score)**	2.79	2.07 to 3.78	< 0.0001	3.06	2.27 to 4.12	< 0.0001
**TV**	0.62	0.55 to 0.71	< 0.0001	0.90	0.78 to 1.05	0.1851
**Radio**	1.22	1.04 to 1.43	0.0165	1.18	1.00 to 1.40	0.0487
**Newspaper**	0.72	0.60 to 0.86	< 0.0004	0.86	0.71 to 1.05	0.1374
**Poster**	1.04	0.92 to 1.18	0.5118	1.18	1.03 to 1.35	0.0145
**Leaflets**	0.93	0.76 to 1.14	0.5086	0.99	0.81 to 1.24	0.9868
**Health worker**	1.70	1.50 to 1.93	< 0.0001	1.65	1.44 to 1.88	< 0.0001
**Volunteer**	1.20	1.04 to 1.38	0.0115	1.05	1.91 to 1.23	0.4947
**Hehaho**	1.41	1.28 to 1.65	< 0.0001	1.26	1.12 to 1.42	0.0002

*adjusted for age and sex of head of household.

^†^adjusted for age and sex of head of household, type of place of residence, household wealth index and household size.

### Effect modification

There was no effect modification by sex of household head for any of the messages, with similar odds of having a child sleep under a net the previous night (Table 3). However, there was a statistically significant effect modification for the composite message by type of place of residence (urban/rural) (p for interaction = 0.009) with composite (any) messages having a greater effect in people living in urban vs. rural areas (OR = 8.58 in urban vs. 2.34 in rural). There was also some evidence for effect modification by type of place of residence for households that had heard a radio message (OR = 1.08 in urban vs. 1.45 in rural; p for interaction = 0.08). Details are shown in Tables 3 and 4.

**Table 3:** Adjusted stratum-specific estimates for sex of household head

**Source of malaria messages**	**Number of children who slept under a net (≥ 1 child vs. no child)**		
	**Female**	**Male**	p-value*
	**OR (95% CI)**	**OR (95% CI)**	
**Any (composite score)**	2.68 (1.92, 3.73)	4.41 (1.96, 9.91)	0.2596
**TV**	1.11 (0.94, 1.31)	1.13 (0.85,1.51)	0.9194
**Radio**	1.36 (1.14, 1.63)	1.33 (0.99, 1.79)	0.8813
**Newspaper**	1.12 (0.92, 1.37)	1.11 (0.75, 1.64)	0.9669
**Poster**	1.25 (1.09, 1.44)	1.41 (1.11, 1.80)	0.3841
**Leaflets**	1.27 (1.02, 1.58)	1.18 (0.81, 1.73)	0.7473
**Health Worker**	1.67 (1.47, 1.91)	2.13 (1.65, 2.74)	0.0975
**Volunteer**	1.42 (1.22, 1.65)	1.69 (1.29, 2.22)	0.2724
**Hehaho**	1.43 (1.25,1.63)	1.55 (1.23, 1.97)	0.5543

*Breslow Day test of homogeneity of ORs.

**Table 4:** Adjusted stratum-specific estimates for type of place of residence

**Source of malaria messages**	**Number of children who slept under a net (≥ 1 child vs. no child)**		
	**Urban**	**Rural**	p-value*
	**OR (95% CI)**	**OR (95% CI)**	
**Any (composite score)**	8.58 (3.19,23.08)	2.34 (1.69,3.22)	0.0089
**TV**	1.01 (0.77,1.32)	1.17 (0.98,1.39)	0.3664
**Radio**	1.08 (0.81,1.45)	1.45 (1.23,1.76)	0.0764
**Newspaper**	1.03 (0.81,1.32)	1.22 (0.94,1.58)	0.3585
**Poster**	1.30 (1.04,1.61)	1.28 (1.10,1.48)	0.9209
**Leaflets**	1.22 (0.94,1.59)	1.28 (0.97,1.67)	0.8231
**Health Worker**	1.80 (1.46,2.23)	1.75 (1.52,2.02)	0.8267
**Volunteer**	1.46 (1.15,1.85)	1.48 (1.26,1.73)	0.9296
**Hehaho**	1.45 (1.20,1.81)	1.45 (1.26,1.67)	0.9088

*Breslow Day test of homogeneity of ORs.

## Discussion

In this nationally-representative cross-sectional study, the proportion of children under five years that slept under a net the previous night was 15.5% and the proportion of households that own a net was 45%. Although this may not be a direct indicator of the hypothesized gap between ITN ownership Vs usage, and “access to a net” that has recently been proposed to replace “household owns at least one net” for use by the Roll Back Malaria’s Monitoring and Evaluation Reference Group (RBM MERG) [13], the data reported here seem to be supportive of such a gap. Using survey data from 11,778 households in Ghana, this study reports a significant positive effect between hearing malaria messages in the electronic/print media and from health workers and usage of bed nets by children under five in a household, with effect sizes ranging between 18-65%. The most effective form of receiving a malaria-related message was from a health worker and the dedicated radio programme “Hehaho’.

Radio is the medium with the widest coverage in Ghana, even in rural areas, as confirmed also by the study results, and hearing a malaria message on the radio was associated with an odds ratio of 1.18 (p = 0.05) for children under five sleeping under a net the previous night. Hearing the dedicated radio programme “Hehaho” was associated with an odds ratio of 1.26 (p = 0.0002) indicating that both radio messages, but especially dedicated radio programmes have an important role in public health education and should be further enhanced. Estimates from a recent study on the impact of a mass media campaign on bed net use in Cameroon [14], also indicated that exposure to the campaign message was associated with an increased odds of 1.68 for children under 5 sleeping under nets the night before.

The use of posters is common and a very frequent site in Ghana [15] and this could be the possible explanation why having read a malaria message from a poster was found to be positively associated with children in a household sleeping under a bed net the previous night (OR = 1.18, p = 0.0145). Even though TV, newspaper and leaflets are also frequently accessed in Ghana, this study reports non-significant effect of messages heard from these media and children sleeping under a net the night before the survey. Perhaps, exposure to these messages is affected by the level of education of the respondent or household head. However, since this was not accounted for, it presents a limitation of the study on the extent of explanation that could be given for the observed non-significance.

Health workers were one of the most effective ways of relating malaria messages, perhaps unsurprisingly. Such workers are sometimes stationed in these areas so the community has better access to information and messages they carry. Although the cost associated with health worker visits, and to a lesser extent with volunteers, is large [16], they were shown here to be more effective in educating people to use bed nets. The finding of a non-significant effect of hearing malaria messages from community volunteers on the odds of children sleeping under a net in this survey contradicts that of another study which reported an association between community intervention programmes involving visits by trained community members and usage of ITN by children under-five years of age [5]. Associations tested here were at the household level (i.e.by asking a representative of the household) and may not necessarily reflect the exposure in other members of the household. It is likely that interventions specially designed for individuals, like the use of mobile phone technology in delivering health messages may be even more effective, but this has to be tested in future studies.

There was statistically significant effect modification by type of place of residence for the composite (any of) malaria message only, with higher odds of having one or more children sleep under a net in urban vs. rural areas (8.59 vs. 2.34; p for interaction =0.009). Although, there is no convincing explanation for this difference based on the individual messages and we cannot exclude the possibility that it may be a spurious finding, it is possible that people in urban areas were exposed to more of the individual messages and perhaps to a greater extent than those living in rural areas thus resulting in a greater effect for any one of the messages in those areas. However, the analysis was limited by the fact that data collected during the survey did not capture frequency/intensity of exposure hence making it impossible to test about the level of exposure.

It is important to also note other limitations of this study. As with all secondary analyses, there were factors that were not accounted for in this study. The proportion of the population that slept under an ITN the previous night as an indicator that defines the behavioral gap in ITN use [13] was not calculated and reported in this study. This is due in part to the fact that this indicator may be biased by seasonality of survey data collection. Furthermore, the use of mosquito coils, a form of repellent could be a contributing reason for people or households not using a net, as pointed out by Buame and Franca-Koh [17]. They asserted that it was important to dispel the idea that nets are not needed if you burn coils (that have a lower cost), pointing to the reason that households opt for coils instead of the bed net. The use of trap doors in households to control mosquito movement is also one reason occupants of households may end up not using a bed net. These reasons may not affect whether someone listened to the malaria related messages or not, but they were not accounted for in the analysis because questions relating to them were not included in the questionnaire used in collecting the data used for this study. Therefore, the major disadvantage was inability to control for other factors that were conceptualized to be potential confounding factors such as those related to attitude and knowledge of how mosquitoes transmit malaria parasite and hence the need to use bed nets among other preventive measures.

The major strength of this study is the sample size. With this large sample size, very narrow confidence intervals as well as sufficient power to test our hypotheses were obtained. The p values shown make chance an unlikely finding and although bias cannot be ruled out in the way the information was gathered (i.e. questionnaires). In addition to that, data used were derived from a survey with nationwide coverage and representativeness taking into account socioeconomic factors like type of place of residence among others. Finally, compared to other studies that have suggested the use of communication and sensitization tools to increase net utilization [2,18-21], this evaluation was of a wider spectrum and perhaps more relevant to available resources, as respondents were asked to recall any messages they might have heard in the past 12 months from eight different sources allowing comparison of different types of messages from a wider national campaign.

### Implications

To reduce malaria infections, it is necessary not only to have bed nets but to also use them. Results from this study suggest that such efforts to relate ITN messages to the public are very useful in increasing use of nets and having multiple ways of reaching the public increases the effect to a large extent. Hearing such messages from health workers and volunteers, as well as from a dedicated radio programme was the most effective way of relating them to the public. Finally, while ways of increasing household’s ownership of ITNs is essential and should also be put into place, awareness campaigns regarding their use are still very important as it has been shown that net ownership does not equal its usage.

## Competing interests

The authors declare that they have no competing interest.

## Authors’ contributions

EOA and AP conceived the study and drafted the manuscript. EOA performed the statistical analysis. All authors read and approved the final manuscript.

## Supplementary Material

Additional file 1 **Associations between socio-demographic characteristics and malaria messages.** The data provided represent the results of statistical analysis showing that the confounders have satisfied all requirements of a confounder. Click here for file

Additional file 2 **Associations between malaria related messages and number of children sleeping under a net the previous night for households with children only.** This table shows the ORs for messages heard and children sleeping under a net the previous night in only households that have children. Click here for file
