# Supplementary material for: Impact of malaria related messages on insecticide-treated net (ITN) use for malaria prevention in Ghana
Source: Malar J. 2014 Mar 28;13:123. doi: 10.1186/1475-2875-13-123 (PMC3997841; doi:10.1186/1475-2875-13-123)
Supplement: Additional file 2 — Associations between malaria related messages and number of children sleeping under a net the previous night for households with children only. This table shows the ORs for messages heard and children sleeping under a net the previous night in only households that have children. [file 1475-2875-13-123-S2.docx]

**Table 1: Associations between malaria related messages and number of children sleeping under a net the previous night in households with children (n=4812)**

|  | **Number of children who slept under a net (** **≥ 1 child vs. No child**) | | | | | |
| --- | --- | --- | --- | --- | --- | --- |
| **Type of malaria message** | **crude OR*** | **95 % CI** | **p-value** | **adjusted OR**† | **95 % CI** | **p-value** |
|  |  |  |  |  |  |  |
| **Any (composite score)** | 2.71 | 1.96 to 3.74 | <0.0001 | 3.00 | 2.17 to 4.14 | <0.0001 |
| **TV** | 0.68 | 0.58 to 0.79 | <0.0001 | 1.88 | 0.74 to 1.04 | 0.1369 |
| **Radio** | 1.38 | 1.15 to 1.65 | 0.0006 | 1.34 | 1.12 to 1.62 | 0.0019 |
| **Newspaper** | 1.03 | 0.83 to 1.28 | 0.8137 | 1.14 | 0.91 to 1.43 | 0.2490 |
| **Poster** | 1.11 | 0.95 to 1.29 | 0.1859 | 1.19 | 1.02 to 1.38 | 0.0317 |
| **Leaflets** | 1.02 | 0.79 to 1.30 | 0.9074 | 1.04 | 0.81 to 1.34 | 0.7419 |
| **Health worker** | 1.38 | 1.19 to 1.61 | <0.0001 | 1.40 | 1.20 to 1.63 | <0.0001 |
| **Volunteer** | 1.19 | 1.00 to 1.41 | 0.0512 | 1.12 | 0.94 to 1.33 | 0.2201 |
| **Hehaho** | 1.39 | 1.21 to 1.59 | <0.0001 | 1.31 | 1.14 to 1.51 | 0.0001 |

* adjusted for age and sex of head of household

†adjusted for age and sex of head of household, type of place of residence, household wealth index and household size
